# A new nomenclature for the livestock-associated *Mycobacterium tuberculosis* complex based on phylogenomics

**DOI:** 10.12688/openreseurope.14029.2

**Published:** 2021-12-01

**Authors:** Michaela Zwyer, Cengiz Çavusoglu, Giovanni Ghielmetti, Maria Lodovica Pacciarini, Erika Scaltriti, Dick Van Soolingen, Anna Dötsch, Miriam Reinhard, Sebastien Gagneux, Daniela Brites

**Affiliations:** 1 University of Basel, Basel, Switzerland; 2 Swiss Tropical and Public Health Institute, Basel, Switzerland; 3 Department of Medical Microbiology, Ege University Faculty of Medicine, Izmir, Turkey; 4 Institute for Food Safety and Hygiene, Section of Veterinary Bacteriology, University of Zurich, Zurich, Switzerland; 5 National Reference Centre for Bovine Tuberculosis, Istituto Zooprofilattico Sperimentale della Lombardia e dell’Emilia Romagna, Brescia, Italy; 6 Risk Analysis and Genomic Epidemiology Unit, Istituto Zooprofilattico Sperimentale della Lombardia e dell'Emilia-Romagna, Parma, Italy; 7 National Institute for Public Health and the Environment (RIVM), Bilthoven, Netherlands Antilles; 8 Department of Medical Microbiology, Radboud University Nijmegen Medical Centre, Nijmegen, The Netherlands

**Keywords:** zoonotic tuberculosis, genetic diversity, mycobacterium tuberculosis complex, phylogenetics, whole-genome sequencing

## Abstract

**Background**: ****The bacteria that compose the *Mycobacterium tuberculosis* complex (MTBC) cause tuberculosis (TB) in humans and in different animals, including livestock. Much progress has been made in understanding the population structure of the human-adapted members of the MTBC by combining phylogenetics with genomics. Accompanying the discovery of new genetic diversity, a body of operational nomenclature has evolved to assist comparative and molecular epidemiological studies of human TB. By contrast, for the livestock-associated MTBC members, *Mycobacterium bovis*, *M. caprae* and *M. orygis*, there has been a lack of comprehensive nomenclature to accommodate new genetic diversity uncovered by emerging phylogenomic studies. We propose to fill this gap by putting forward a new nomenclature covering the main phylogenetic groups within *M. bovis*, *M. caprae* and *M. orygis*.

**Methods**: ****We gathered a total of 8,736 whole-genome sequences (WGS) from public sources and 39 newly sequenced strains, and selected a subset of 829 WGS, representative of the worldwide diversity of *M. bovis*, *M. caprae* and *M. orygis*. We used phylogenetics and genetic diversity patterns inferred from WGS to define groups.

**Results**: ****We propose to divide *M. bovis*, *M. caprae* and *M. orygis* in three main phylogenetic lineages, which we named La1, La2 and La3, respectively. Within La1, we identified several monophyletic groups, which we propose to classify into eight sublineages (La1.1-La1.8). These sublineages differed in geographic distribution, with some being geographically restricted and others globally widespread, suggesting different expansion abilities. To ease molecular characterization of these MTBC groups by the community, we provide phylogenetically informed, single nucleotide polymorphisms that can be used as barcodes for genotyping. These markers were implemented in KvarQ and TB-Profiler, which are platform-independent, open-source tools.

**Conclusions**: ****Our results contribute to an improved classification of the genetic diversity within the livestock-associated MTBC, which will benefit future molecular epidemiological and evolutionary studies.

## Plain language summary

Tuberculosis affects humans and livestock species. Its etiological agents are different bacteria belonging to the *Mycobacterium tuberculosis* complex (MTBC). In recent years, whole-genome sequencing (WGS) has become essential in both basic and clinical tuberculosis research. Based on WGS, different human-adapted MTBC genotypes have been classified into lineages and sublineages, which have been shown to differ in their geographic distribution and in virulence. Studies based on WGS are starting to emerge also for livestock-associated MTBC pathogens, but an overarching operational nomenclature systematically covering all known genetic diversity is missing. After gathering several thousands of WGS, we propose here a backbone of operational nomenclature to classify the genetic diversity uncovered by genomic studies of livestock-associated MTBC. Furthermore, a set of molecular markers are provided which can be used to identify the newly proposed lineages and sublineages.

## Introduction

Tuberculosis is a leading cause of morbidity and mortality in humans ^1^. Moreover, bovine TB (bTB) remains a major economic problem and continues to be a zoonotic threat in many places around the world ^2, 3^. Human TB is caused mostly by members of the *Mycobacterium tuberculosis* complex (MTBC) collectively known as *Mycobacterium tuberculosis,* and *M. africanum*, whereas bTB in livestock is primarily caused by *M. bovis*. These organisms, and the other members of the MTBC ^4^, share more than 99% identical nucleotide sequences but can vary considerably in gene content ^5^. The MTBC comprises several unique phylogenetic lineages that differ mostly by chromosomal deletions and point mutations. No significant homologous recombination between strains or gene insertion via horizontal gene transfer occurs in the MTBC ^6– 8^. Despite their high genetic similarity and strict clonality, these lineages exhibit striking differences in host tropism, infecting a wide range of mammalian hosts ^9^. For the human-adapted MTBC, a good understanding of the population structure has emerged through comparative analyses of whole-genome sequences (WGS) of TB patient isolates from all over the world. The human-adapted MTBC can be classified into nine phylogenetic lineages: Lineage 1 (L1) to L7, and more recently, two new lineages, L8 ^10^ and L9 ^11^, have been described but remain poorly characterized. Lineages 1-4 and L7 correspond collectively to *M.tuberculosis sensu stricto*, whereas L5 and L6 are traditionally known as *M. africanum*. Further subdivisions among the human-adapted MTBC lineages have been proposed by many different studies to highlight existing within-lineage differences in geographic distribution and genetic differentiation. By contrast, the animal-adapted members of the MTBC remain much less well characterized, and are typically named according to the host species from which they were first, or most commonly, isolated. Considering the growing number of WGS available for many of these pathogens, a more comprehensive and systematic nomenclature beyond the species name is necessary for assisting comparative and molecular epidemiology studies. This is of most relevance for those animal-adapted MTBC members which are a significant cause of TB in livestock species and which also have a high zoonotic potential. In this study, we considered as livestock-associated those MTBC lineages whose evolutionary success is linked to their ability to cause infection and transmit within livestock populations in addition to other host species; *M. bovis*, *M. caprae* and *M. orygis*. Occasionally, TB in livestock can be caused by *M. tuberculosis sensu stricto* or *M. microti*, but these members of the complex have not been shown to transmit within livestock. The low virulence of *M. tuberculosis sensu stricto* in cattle compared to M. bovis has also been demonstrated in experimental infections of cattle ^12^.

For *M. bovis*, there are currently thousands of WGS in the public domain. However, until recently, genetic diversity of *M. bovis* populations was described based on four major groups of genotypes defined by genomic deletions and SNPs. These groups were known as clonal complexes European 1 and 2 (Eu1 and Eu2), and African 1 and 2 (Af1 and Af2). The study of these clonal complexes brought major insights into the genetic diversity underlying bTB in Europe, the Americas and New Zealand (Eu1 and Eu2), as well as in West- and East Africa (Af1 and Af2, respectively) ^13– 16^. More recently, we and others, have gathered several thousands WGS of M. bovis, generating initial insights into the world-wide population structure of this pathogen based on complete genomes ^17– 21^. Through these efforts, several *M. bovis* sub-populations were identified, and while some corresponded to the previously identified clonal complexes ^13– 16, 21^, several others remained unclassified ^17– 21^.

Whereas *M. caprae* is a known cause of infection in livestock species, the association of *M. orygis* with livestock infections is less well established. *M. orygis*, initially thought to be a pathogen of antelope species, has in the meantime been isolated from different hosts ^22– 24^. Importantly, most available strains today were isolated from humans of South Asian origin ^25– 30^. In South Asia, *M. orygis* has recently been proposed to be the main cause of zoonotic TB ^30^. The main reservoirs of *M. orygis* remain poorly understood, yet it has been isolated from cattle in India and Bangladesh ^23, 25^, and also shown to actively transmit within cattle ^23^. India is the country with the biggest cattle population of the world, often living in close proximity with humans, favoring the hypothesis that livestock is the most likely source of zoonotic infections caused by *M. orygis*. Due to its high zoonotic prevalence, the number of *M. orygis* WGS available is steadily increasing, which urges for new definitions aiding comparative genomics.

Here, we propose a comprehensive nomenclature, based on phylogenetic principles and genetic diversity patterns, for the main groups found in what is currently known as *M. bovis*, *M. caprae* and *M. orygis*. The nomenclature used for the different members of the MTBC has been repeatedly revised over time, with a particular focus on whether the different MTBC members should be considered separated species or the same species given their high genomic similarity ^31^. Classifying the different MTBC members into ecotypes has also been proposed, to better accommodate the differences in host range of the different MTBC members ^32, 33^. The nomenclature we propose here is not intended as a replacement but rather to serve as an operational nomenclature to assist genomic comparative studies. We propose to take the same hierarchical levels of classification as has been adopted for the human-adapted MTBC lineages and sublineages, as it has proven to be robust and flexible enough to capture diversity both at a global and local level, and is also adequate to describe newly discovered diversity (e.g. L9 and L8). Given the difficulties in defining populations in bacteria, we would like to emphasize that the nomenclature proposed here, might, but does not necessarily have to reflect cohesive groups sharing biological properties. It is rather a pragmatic attempt to find a classification that will usefully describe the genetic diversity and the phylogeographic patterns observed in the MTBC affecting livestock.

## Methods

### Data collection

***Representative dataset for livestock-associated MTBC.*** We searched the US National Center for Biotechnology Information (NCBI) for new publicly available WGS of *M. bovis*, *M. caprae* and *M. orygis*, using names as search terms: for example for *M. bovis,* “ *Mycobacterium tuberculosis variant bovis* [organism]” was searched. Our search was restricted to the time period between the 11 ^th^ of March 2019, when we already had gathered 3,364 WGS ^17^, until the 4 ^th^ of November 2020. A total of 5,383 new genomes concordant with our search terms were available. From these genomes, we excluded those that met the following criteria prior to analysis: genomes registered as bacillus Calmette-Guérin (BCG), as laboratory strains, with unknown country of isolation or isolated in countries already over-represented in previous analyses (Mexico, USA, UK, New Zeeland) ^17^, and genomes corresponding to strains isolated in patients from low endemic countries with unknown country of origin. Genomes that were publicly available but unpublished at the time of WGS retrieval, were also excluded after a preliminary analysis, as they did not provide new main phylogenetic clades once compared to the representative set of genomes of *M. bovis* and *M. caprae* previously published ^17^. Finally, WGS that did not meet our criteria for downstream analysis (average whole-genome coverage > 15x and ratio of heterogenous SNPs to fixed SNPs < 1) were excluded. Furthermore, we newly sequenced 19 genomes from Turkey isolated from humans, two genomes from Italy isolated in cattle in Apulia and Sicily ^34^, and four genomes from Switzerland isolated in cattle ^35^. The selected genomes were added to a previous reference set representing the world-wide diversity of *M. bovis* (n=464) and *M. caprae* (n=12) selected after an initial compilation of 3,364 WGS ^17^. For *M. orygis*, 14 newly sequenced genomes isolated from patients and from different zoo animals of South Asian origin ^22^ were obtained and analysed together with 77 publicly available WGS ( *Extended data*, Table 1). In total, 829 representative genomes were considered, of which 675 were *M. bovis*, 63 *M. caprae*, and 91 *M. orygis*. With respect to our previous representative dataset ^17^, 211 new genomes were added to the downstream analysis for *M. bovis* ( *Extended data*, Table 1). Most of these were from animal strains isolated in Brazil (n=19), France (n=83), Germany (n=40), Ethiopia (n=37) and Mali (n=3) ^18– 21, 36^, while few derived from human isolates from Tanzania (n=1), Indonesia (n=1), Kazakhstan (n=2) and Moldova (n=1) ^37– 39^ ( *Extended data,* Table 1). In the case of *M. caprae*, 51 genomes isolated from Spain were added ^40^. The 39 newly sequenced genomes were uploaded to EBI under the study accession numbers PRJEB46653 and PRJEB46575 ( *Extended data,* Table 1).

***Representative dataset for the complete MTBC.*** In order to obtain a representative set of world-wide sampled MTBC genomes from both animal and human isolates with a discernible tree topology, we randomly selected genomes from a large in-house collection of WGS (approximately 50,000), for the human lineages 1-6 and for *M. bovis*. The genomes were selected according to the following scheme: 50 random genomes per continent (Africa, America, Asia, Europe, and Oceania) for each lineage. For lineage 1-6, genomes isolated in Northern America, Europe (except Eastern Europe), and Oceania were required to have information about the country of birth of the patient to be considered. Furthermore, WGS from the following strains were added: three strains belonging to the proto Beijing sublineage, eight pyrazinamide susceptible *M. bovis* strains ^17^, five L9 strains, 23 L7 strains, two L8 strains, 57 *M. caprae* strains, 15 *M. microti* strains, 84 *M. orygis* strains, six *M. pinnipedii* strains, two ancient genomes from Peruvian mummies, one each of Chimp and Dassie bacillus, and one each of *M. mungi* and *M. suricattae*. A complete list containing the accession numbers of all genomes included (n=1,221) can be found in the supplementary data ( *Extended data*, Table 2).

### Bacterial culture, DNA extraction and whole-genome sequencing

The MTBC isolates were grown in 7H9-Tween 0.05% medium (BD) +/- 40mM sodium pyruvate. We extracted genomic DNA after harvesting the bacterial cultures in the late exponential phase of growth using the CTAB method ^41^. Sequencing libraries were prepared using NEXTERA XT DNA and the EBNext Ultra II DNA Library Preparation Kits (Illumina, San Diego, USA). Multiplexed libraries were paired-end and single-end sequenced using Illumina HiSeq 2500 (Illumina, San Diego, USA), Illumina NovaSeq 6000 (Illumina, San Diego, USA) and MiSeq (Illumina, San Diego, USA) with 151, 101 and 250 cycles, respectively.

### Bioinformatic analysis

***Whole-genome sequence analysis.*** All WGS downloaded, as well as those generated in-house, were analyzed using the WGS analysis pipeline described in 42. Briefly, the retrieved FASTQ files were processed with Trimmomatic v0.3 ^43^ to remove the Illumina adaptors and to trim low quality reads. Only reads of at least 20 bp were kept for further analysis. SeqPrep v 1.2 was then used to merge overlapping paired-end reads (overlap size = 15). We then mapped the resulting reads using BWA v0.7.13 ^44^ (mem algorithm). with respect to the chromosome of the *M. tuberculosis* H37Rv (NC_000962.3, NCBI). As a reference sequence, we used a reconstructed ancestral sequence of the MTBC ^45^ where at each position of the chromosome NC_000962.3 the inferred nucleotide of the ancestor of MTBC is the reference. Duplicated reads were marked by the Mark Duplicates module of Picard v 2.9.1 and then excluded. We further performed local realignment of reads around INDELs using the RealignerTargetCreator and IndelRealigner modules of GATK v 3.4.0 ^46^. Samtools v1.2 mpileup ^47^ and VarScan v2.4.1 ^48^ were then used for SNP calling with the subsequent thresholds: minimum mapping quality of 20, minimum base quality at a position of 20, minimum read depth at a position of 7x and maximum strand bias of 90%. Only SNPs with a frequency of ≥ 90% within an isolate were considered, and for those with a frequency of ≤ 10% the ancestor state was called. The *M. tuberculosis* H37Rv reference annotation (NC_000962.3, NCBI) was used as the reference genome of *M. bovis* (AF2122/97, NCBI) has no genes absent from H37Rv, except for TbD1 ^49^. SNPs were annotated with SnpEff v4.11 ^50^. Positions falling in PE/PPE genes, phages, insertion sequences, and in regions with at least 50 bp identity to other genomic regions were excluded ^51^.

***In silico spoligotyping.*** All WGS were *in silico* spoligotyped using KvarQ ^52^. The respective SB numbers were retrieved by entering the spoligotype patterns into the *Mycobacterium bovis* Spoligotype Database and are reported in *Extended data*, Table 1.

***Phylogenetic analyses.*** The phylogenetic trees were constructed from alignments of variable positions with a percentage of missing data of ≤ 10%. With RAxML v 8.2.11 ^53^ maximum-likelihood phylogenies were constructed by using the general time-reversible model of sequence evolution (-m GTRCAT –V), a rapid bootstrap analysis with 1000 bootstraps and search for the best-scoring maximum-likelihood phylogeny. The MTBC phylogeny was rooted with *M. canetti* (SAMN00102920, NCBI) while all other phylogenies were rooted with a MTBC lineage 6 strain (SAMEA3359865, NCBI). Phylogenetic trees were plotted with ggtree ^54^ and Figtree.

***Population structure and genetic distances.*** Population structure was evaluated using a Principal Component Analysis (PCA) based on all polymorphic positions obtained from the 1,221 dataset,using the R package adegenet ^55^ in R 3.5.2. Between and within group genetic distances were measured as raw pair-wise SNP differences for the different groups using the R package ape ^56^.

***Maps of geographic distribution.*** The geographical origin of the isolates and host-related metadata were recovered from NCBI and used to inform geographic ranges. Strains isolated from zoo animals or isolated from humans living in Europe, Oceania, or North America with unknown place of birth were not taken into account. Since WGS is not performed on a regular basis in all countries, relying only on WGS data would underestimate the geographical distribution of certain clades. To adjust for that, we used the *in silico* SB numbers shown to be phylogenetically informative ^17^, and searched for publications reporting those SB numbers and their associated geography ( *Extended data*, Table 3). The countries of isolation identified in this way were added to those obtained from the WGS and were used to obtain geographic distributions using the rworldmap package ^57^ in R 3.5.2 ^58^.

***Validation of lineage- and sublineage- specific markers.*** In order to obtain a list of polymorphic positions specific to all members of a defined lineage or sublineage, the variant calls obtained from the 829 La1, La2, and La3 WGS were merged using BCFtools. On the merged dataset, the following filtering steps were applied: First only positions mutated in at least seven genomes were kept using VCFtools (--mac 7) ^59^, second only positions with a FILTER flag PASS were kept using BCFtools. The first filtering step was included, since we were only interested in SNPs that were common to all members of a sublineage and the lowest number of WGS for a sublineage was seven (unknown6). A genotype matrix was created using the R package VariantAnnotation ^60^ and by using customized python scripts, those variants mutated in all members of a specific lineage or sublineage, or in a monophyletic group of multiple sublineages (e.g. La1.3 and La1.2) were extracted. This resulted in a list containing 2,203 variants specific to 19 different sublineages and combinations of multiple sublineages. Additionally, we created a list of polymorphic positions using 4,742 WGS representing the genetic diversity of human-adapted lineages L1-L7 and L9 ^42^ and to ensure that our SNPs defining lineages and sublineages among livestock-associated MTBC were specific, we excluded all positions that were polymorphic in the set of 4,742 genomes. This way, a final list of 1,959 SNPs specific to a lineage, sublineage or sublineage combinations within livestock-associated MTBC, and not polymorphic in any of human-adapted MTBC lineages, was generated ( *Extended data*, Table 4). Out of the 1863 SNPs, 80 (two to five variants per lineage and sublineage or sublineage combinations) were selected to create a new test suite ^61^ specific for the livestock-associated MTBC in KvarQ ^52^. In order to validate the specificity of the 87 SNPs used in the new KvarQ test suite, we scanned 2,861 livestock-associated WGS from Loiseau *et al*. 2020 ^17^ that were not included in the 829 dataset, and 66 additional WGS randomly chosen from recent publications ^62– 64^ ( *Extended data*, Table 5). The 2,927 fastq files were also processed using the workflow described in the WGS sequence analysis section and a phylogenetic tree was inferred as described above. The phylogenetic tree was compared to the lineage and sublineage identity as determined by KvarQ, to assess the accuracy and specificity of the test suite.

## Results and discussion

### Classification of livestock-associated MTBC into new lineages

**Figure 1.**
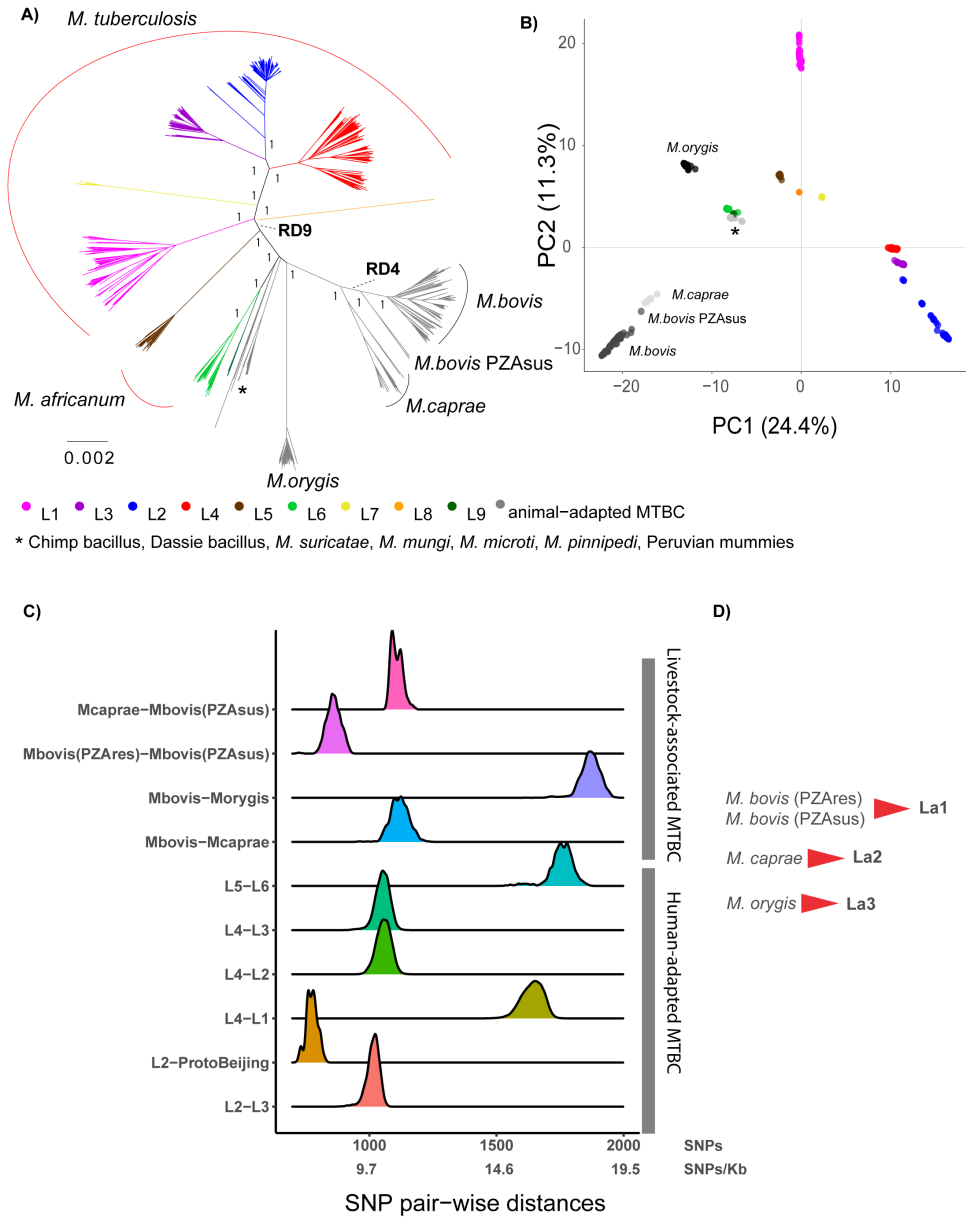
**A**) Maximum Likelihood topology of 1,221 MTBC representatives, where 50 representatives were randomly selected from each continent and from each lineage (see methods). The tree was inferred from an alignment containing 103,843 polymorphic positions. Branch lengths are proportional to nucleotide substitutions. Support values correspond to bootstrap values. Members of the human-adapted MTBC have tips colored according to their lineage. **B**) Principal Component Analysis (PCA) derived from the same alignment as the phylogeny. The two first principal components are shown. **C**) Distribution of the raw pairwise SNP distances between human adapted MTBC lineages and between different animal adapted MTBC members. **D**) Proposed lineage nomenclature for *M. bovis* susceptible and resistant to pyrazinamide, *M. caprae* and *M. orygis*.

After screening an extensive collection of approximately 50,000 WGS, we compiled a comprehensive set of 1,221 WGS representing all MTBC members from all continents in the world (except Antarctica). For the human-adapted lineages (L1 to L6) as well as for *M. bovis*, a large number of WGS is available, and in order to obtain an even representation of these groups with a discernable topology, 50 representatives were randomly selected from each continent and from each lineage. The phylogenetic relationships of these randomly selected 1,221 MTBC strains are represented in Figure 1A. The results indicated that the human-adapted MTBC members are paraphyletic, given that the group defined by the Region of Difference 9 (RD9) ^65^ comprises human (L6 and L9) and animal-adapted members ( Figure 1A), in line with previous findings ^4^. While the distinct clades of the human-adapted members were separated into different lineages and have been named accordingly (Lineage 1-9), the animal-adapted members are still only referred to by their species name. Recent studies, and our searches for WGS from the public domain, indicated that there is a wealth of WGS, in particular for *M. bovis*, representing different geographical areas, hosts, and epidemiological settings of the world ^17– 21, 36, 66– 68^. *M. orygis*, which has been recently suggested to be the main cause of zoonotic TB in South Asia and possibly a pathogen of cattle in that region ^25^, also has a growing number of genomes available. There is, however, a lack of consistent nomenclature to assist in the comparative analysis of these genomes. Therefore, we propose to adopt a lineage nomenclature that covers the main groups found in what is currently known as *M. bovis*, *M. caprae* and *M. orygis* based on phylogenetics and genetic diversity patterns. For the remaining animal-adapted MTBC members, *M. mungi, M. suricattae*, the Dassie and Chimpanzee bacillus, as well as *M. pinnipedii and M. microti*, still too few WGS were available to allow for any meaningful within-lineage diversity analysis. In addition, the host range and ecology of these ecotypes remain poorly understood. We reasoned that these cases would require more extensive sampling, and thus focused the remaining of our analyses on *M. bovis*, *M. caprae* and *M. orygis*.

### A phylogenomics-based nomenclature for *M. bovis*, *M. caprae* and *M. orygis*

These three members of the MTBC evolved from a common ancestor not shared by any other group within the MTBC ( Figure 1A). The visual inspection of the phylogeny and the PCA plot suggested that among these three groups, there are four main phylogenetic clades: *M. orygis*, *M. caprae*, the pyrazinamide-susceptible *M. bovis* ^17^ and the pyrazinamide-resistant *M. bovis* ( Figure 1 A&B). The long branches leading to these clades indicate that many genetic changes have occurred in their founding ancestor populations, and this was also reflected in the pair-wise SNP distances between these clades estimated from the 1,221 whole-genomes dataset ( Figure 1C). We suggest classifying these four clades into three main lineages within the MTBC analogously to the human lineages, considering *M. bovis* pyrazinamide- resistant and -susceptible as one lineage, and *M. caprae* and *M. orygis* as two other main lineages. We propose adopting the numerical lineage nomenclature used for the human-adapted MTBC members, adding the lower-letter “a” standing for “animal *”*. This nomenclature distinguishes the human-adapted from the remaining members of the complex, which can be of relevance for clinicians; simultaneously, for the non-human adapted MTBC members, it has the advantage of being agnostic with respect to the host species, which can be multiple. In this way, we suggest naming La1, La2 and La3 the groups currently known as pyrazinamide-resistant and -susceptible *M. bovis*, *M. caprae* and *M. orygis*, respectively ( Figure 1D).

**Figure 2.**
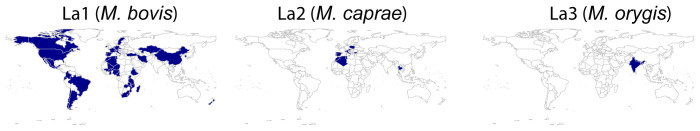
Geographic distribution of La1, La2 and La3 informed by WGS and *in silico* spoligotype patterns.

The pyrazinamide-susceptible *M. bovis* group is composed of pyrazinamide-susceptible strains within *M. bovis*, and is geographically restricted to East Africa ( *Extended data*, Table 1, Figure 2) ^17^. This group of strains were quite divergent from the pyrazinamide-resistant *M. bovis*, yet closer to the latter than to *M. caprae* ( Figure 1C). A similar situation occurred within the human-adapted L2 when comparing the so-called Proto-Beijing group with the remaining strains of L2 ( Figure 1C). The available WGS of pyrazinamide-susceptible *M. bovis* came from strains isolated in humans, cattle and a zoo antelope, and no new WGS in our current analysis have been added with respect to previous studies ^17, 20^. *In silico* determination of spoligotypes ( *Extended data*, Table 1) revealed that similar patterns are common in cattle from Tanzania and Uganda ^15, 69, 70^, and have also been observed in different wild animal species in Tanzania ^70^. In our extensive WGS collections of MTBC isolates from TB patients in Uganda and Tanzania (unpublished), we did not find any representatives of pyrazinamide-susceptible *M. bovis*, suggesting that zoonotic transfers of this group of strains are rare, like for other *M. bovis* strains.

Unlike the human-adapted lineages of the MTBC, La1, La2 and La3 are multi-host pathogens known to infect livestock and other wild mammal species, and occasionally humans ^9, 25^. The multiple host species from which these isolates were obtained, are in line with that notion ( *Extended data*, Table 1). Despite this general broad host range, these lineages differ substantially in their geographic distribution, suggesting local adaptation to different hosts and/or different dispersion abilities of their host populations ( Figure 2). The evolutionary success of La1, and its broad distribution around the world, are linked with the ability of La1 to infect different species of livestock, in particular cattle. Additionally, its broad host tropism also contributes to this success, as demonstrated by the difficulties in eradicating bovine TB even in high-resource countries, where La1 can be maintained in different wildlife species that live in close proximity to livestock such as badgers, deer, or wild boar, or possums ^71^. Various molecular markers, and more recently WGS, suggest no preferential association of La1 genotypes with particular host species ^68, 72, 73^. It is thus still unclear whether La1 infections in non-bovid species are the result of spillover events from cattle populations (i.e. La1 is better adapted to cattle than to other animal species), or if La1 has an intrinsically broader host spectrum that can lead to similarly successful infectious cycles in many different animal species. Interestingly, despite its broad host repertoire and the ability to cause zoonotic TB, La1 is not able to sustainably cause infectious cycles in immune-competent humans. Despite being much less studied, a similar rationale might apply to La2 and La3, as we shall discuss next.

**Figure 3.**
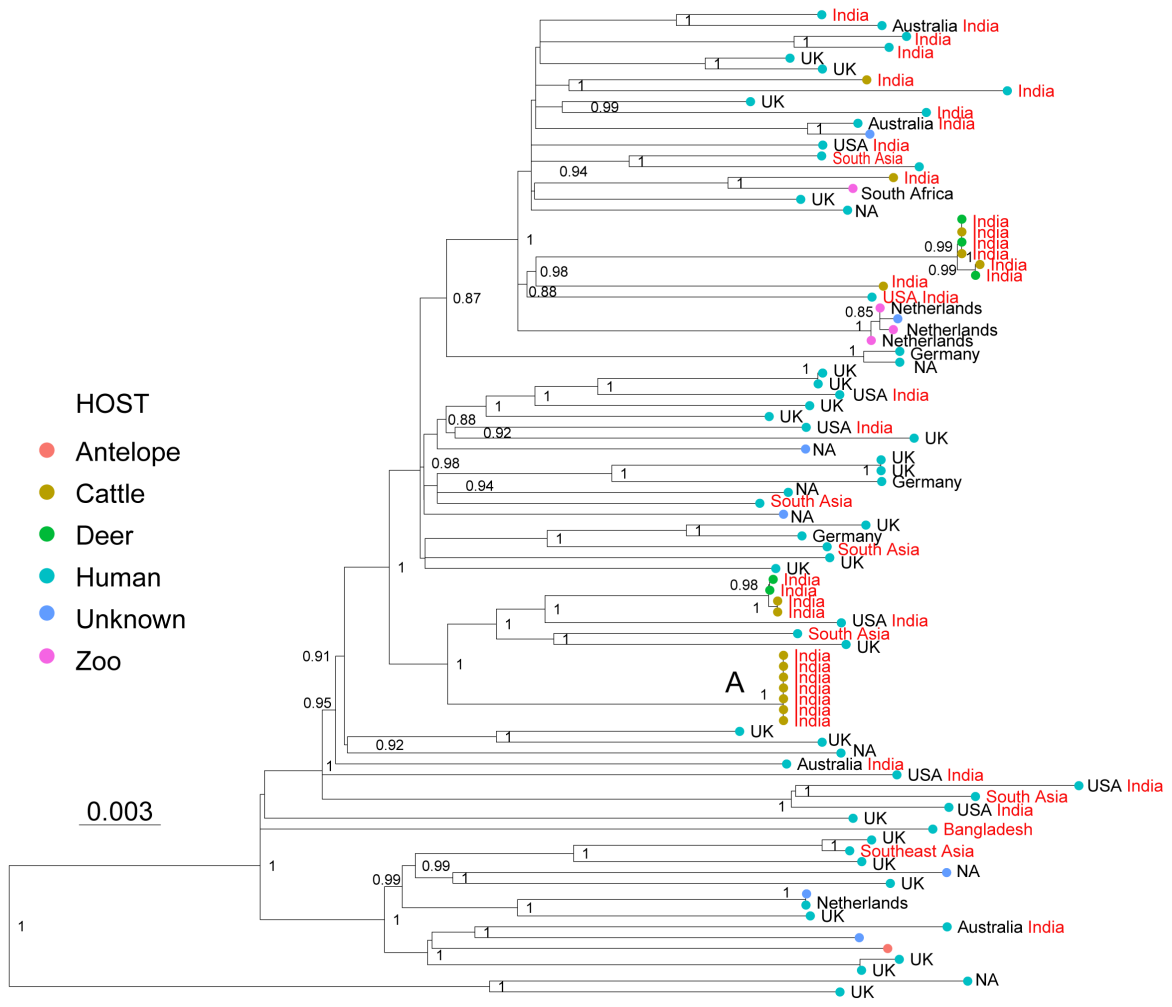
Maximum Likelihood topology based on 2,114 polymorphic positions derived from 91 WGS of La3, after conservatively filtering out several repetitive regions of the genome (see methods). Branch lengths are proportional to nucleotide substitutions and the topology is rooted with one L6 WGS. Support values correspond to bootstrap values. The different colors of the tips correspond to different hosts indicated in the legend. Country of isolation is indicated, followed by the country of birth in the case of human isolates, when known. Isolates with origin in South Asia are indicated in red. A cluster of WGS obtained from cattle isolated is indicated with A.

**Figure 4.**
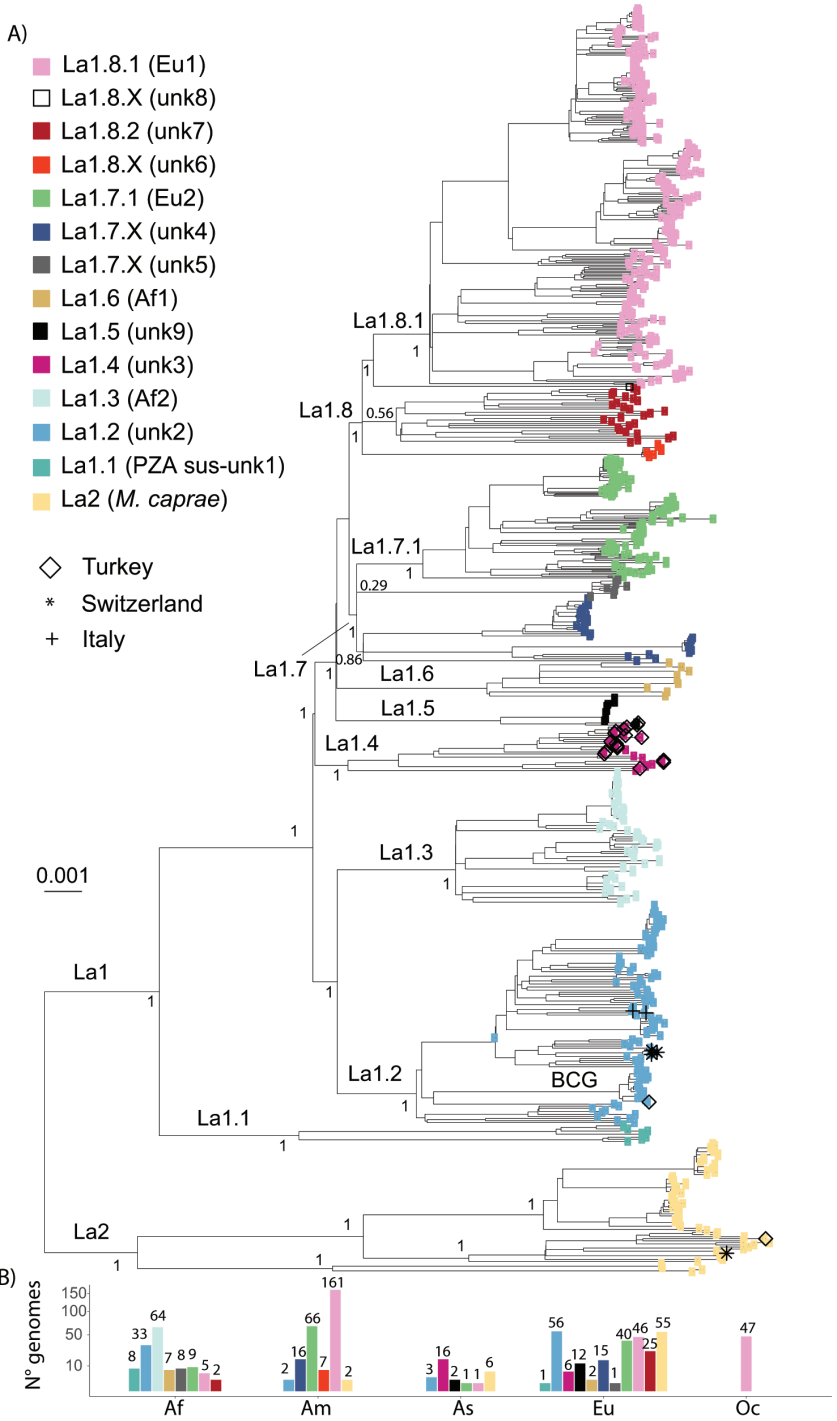
**A**) Maximum Likelihood topology based on 34,308 polymorphic positions derived from 675 La1 and 63 La2 WGS, after conservatively filtering out several repetitive regions of the genome (see methods). Branch lengths are proportional to nucleotide substitutions and the topology is rooted with one L6 WGS. Support values indicated for the main divisions correspond to bootstrap values. Monophyletic clades corresponding to sublineage divisions are indicated in color as in the legend. Newly sequence La1 in this study are indicated by different symbols as in the legend. **B**) Number of WGS included in the phylogenetic tree per continent (Af = Africa, Am = America, As = Asia, Eu = Europe, Oc = Oceania) and sublineage shown on a square-root scale. The bars are colored according to the sublineage.

La2, or *M. caprae*, is globally associated with a much lower burden of disease compared to La1, and that is presumably also reflected in a much lower number of WGS available. La2 is, however, a significant regional cause of animal TB as it is the main cause of TB in goats in the Iberian Peninsula ^74^, affects several livestock and wild animal species populations in Central Europe ^75, 76^, and is occasionally a source of zoonotic TB ^77^. Indeed, a study in Germany showed that up to one third of zoonotic TB cases in that country were caused by La2 ^78^. Two of our newly sequenced genomes belonged to La2, with one corresponding to an isolate from cattle in Switzerland ^35^ and the other from a patient in Turkey ( Figure 4). Both were closely related to La2 strains isolated in Spain and in Germany ^40, 79^. The geographic distribution of La2 obtained from the WGS metadata and from searching the literature using the spoligotypes patterns determined *in silico* ( *Extended data,* Table 3) confirms, as previously suggested, that La2 is not restricted to Europe ^17^ but also occurs in Africa, South America and East Asia ( *Extended data*, Table 1, Figure 2). Our phylogenetic reconstruction also revealed that La2 exhibits strong population divisions, in particular between isolates of Asian and European origin ( Figure 1A & Figure 3). However, better sampling, including more isolates from Africa, America and Asia, will be necessary to better understand the biogeography and evolutionary history of La2.

The most distantly related group within the livestock-associated lineages is La3, commonly known as *M. orygis*. La3 was originally isolated from a captive *oryx* antelope, and has since then been isolated from many different wild, zoo and domestic animals, and from patients of South Asian origin in low endemic TB countries ^22, 26– 29^. In India, Bangladesh and Nepal, La3 has been isolated from humans, cattle, primates, deer and a wild rhinoceros ^23– 25^. The native geographic distribution of this pathogen seems to be restricted to South Asia where it is possibly the main cause of zoonotic TB ^25^. Here, we compiled 91 WGS of La3 from different sources: 1) isolates from low TB endemic countries from patients of South Asian (n=13) or unknown origin (n=35) ^30^, 2) isolates from patients in Southern India (n=5) ^25^ and one patient from Bangladesh ^80^, 3) isolates from cattle (n=15) and deer (n=5) from different Indian regions ^25^. The remaining publicly available genomes were of unknown origin and unknown host species. The 14 newly sequenced La3 isolates were obtained from zoo animals and from patients of South Asian origin in the Netherlands ^22^ ( *Extended data*, Table 1). The genetic relationships among the 91 WGS showed that the isolates from low TB endemic countries, isolates from zoo animals, and isolates obtained in India, both from patients and from veterinarian samples, appeared intermingled in the phylogenetic tree; they were separated by relatively long branches, suggesting a common origin of infection in South Asia ( Figure 3). Little is known about the transmission of La3, and the host preferences of this pathogen also remain unclear ^25, 81^. The phylogenetic relationships presented here are consistent with direct transmission from cattle-to-cattle in India ( Figure 3, cluster A), but they remain inconclusive with respect to direct transmission among and between the other host species. Cattle-to-cattle transmission of La3 inferred through mini-satellite markers (MIRU-VNTR) has been reported previously in Bangladesh ^23^. In contrast, no evidence of patient-to-patient transmission has been shown yet, although transmission from one TB patient to cattle has been reported ^26^, suggesting that humans are not necessarily a dead end for La3. The La3 patient samples analyzed here are not well-suited to capture direct transmission given that they mostly represent active TB cases in emigrated patients who most likely have acquired their infection in their country of origin. One exception was the data published by Duffy and colleagues ^25^, which was the first to report infection in patients by La3 within the endemic geographic range of this pathogen. Their findings suggest that human infections by La3 are relatively rare when compared to *M. tuberculosis*, given that, of the almost 1,000 patient samples collected in a referral hospital in southern India, only 0.7% belonged to La3. In addition, patients reported to be infected with La3 were often associated with non-pulmonary TB ^25, 27^. This is indirect evidence pointing to La3 not being very successful at maintaining infectious cycles in humans, in a way that is reminiscent of zoonotic infections by *M. bovis*, as already suggested by 25. Future studies are needed to better understand the host preferences of La3 and how this lineage is transmitted between species. However, given that bTB is endemic in India, which also harbors the largest population of cattle in the world ^82^, a plausible scenario is that cattle may play an important role in the dynamics of La3 infections.

### Sublineages within La1

Lineage a1 is the most studied member of the animal-adapted MTBC, since bTB has a major economic impact and it is the most common cause of zoonotic TB ^9, 83^. In recent years, several studies have compared large collections of WGS of *M. bovis*, bringing new insights into the local transmission dynamics and into to the global population structure, phylogeography and evolutionary history of this pathogen ^17– 20, 66, 68^. In a previous study, after an initial compilation of 3,364 genomes representing 35 countries around the world, we defined a reference set of 476 WGS representing the global diversity of *M. bovis* (n=464) and *M. caprae* (n=12) ^17^. Our results revealed that a large proportion of these genomes belonged to the clonal complex Eu1 ^13^, reflecting biases in sampling and WGS efforts towards the United Kingdom and its former trading partners. Other regions of the world with high *M. bovis* prevalence remained comparatively under-sampled, and yet, we identified several clades within *M. bovis* that did not belong to either Eu1 or to any of the clonal complexes known at the time ^17^. Here, we aimed to improve the WGS representation of these previously unclassified clades and to identify new clades by including WGS from countries that were previously under-sampled. After a new search of 5,383 entries on the public domain, following a set of exclusion criteria (see M), and our own sequencing efforts (19 strains from Turkey, four from Switzerland and two from Italy), we added 221 *M. bovis* and 63 *M. caprae* WGS to the previous identified reference set of 476 WGS ^17^. In total, we newly analysed 675 La1 and 63 La2 WGS. The phylogenetic reconstruction of the 738 genomes represented in Figure 4 revealed several clades diverging early from the common ancestor of all La1. All these clades were previously identified, and while some formed monophyletic groups corresponding to clonal complexes already defined (Eu1, Eu2, Af1 and Af2), the remaining clades were named transiently as unknown1 to unknown9 ^17, 20^. In the present analysis, several WGS were added to these unclassified clades, but including more WGS available at NCBI (see methods) did not uncover any new deeply rooted and divergent clades. We therefore considered that the 675 genomes selected here provided a good representation of the global diversity of La1 in its main groups, and could be used to delineate a systematic nomenclature to assist future comparative studies.

Branches that represent deep splits from the most recent common ancestor of La1 leading to monophyletic groups represent evolutionary successful populations deriving from common founder events, and thus from a common genetic pool. The strains belonging to these monophyletic groups might share biological properties, which are more similar within than between groups. We have used this rationale to split La1 into several clades, which we hereafter call sublineages in analogy to the human-adapted sublineages of the MTBC. In addition, to increase the operative value of this nomenclature, we have taken into account the geographic distribution of these groups whenever possible, and have attempted to be consistent with the clonal complex nomenclature already in use ^17, 19^. The correspondence between the nomenclature we propose here and previously defined groups based on WGS ^19, 21^ is given in *Extended data*, Table 1.

**Figure 5.**
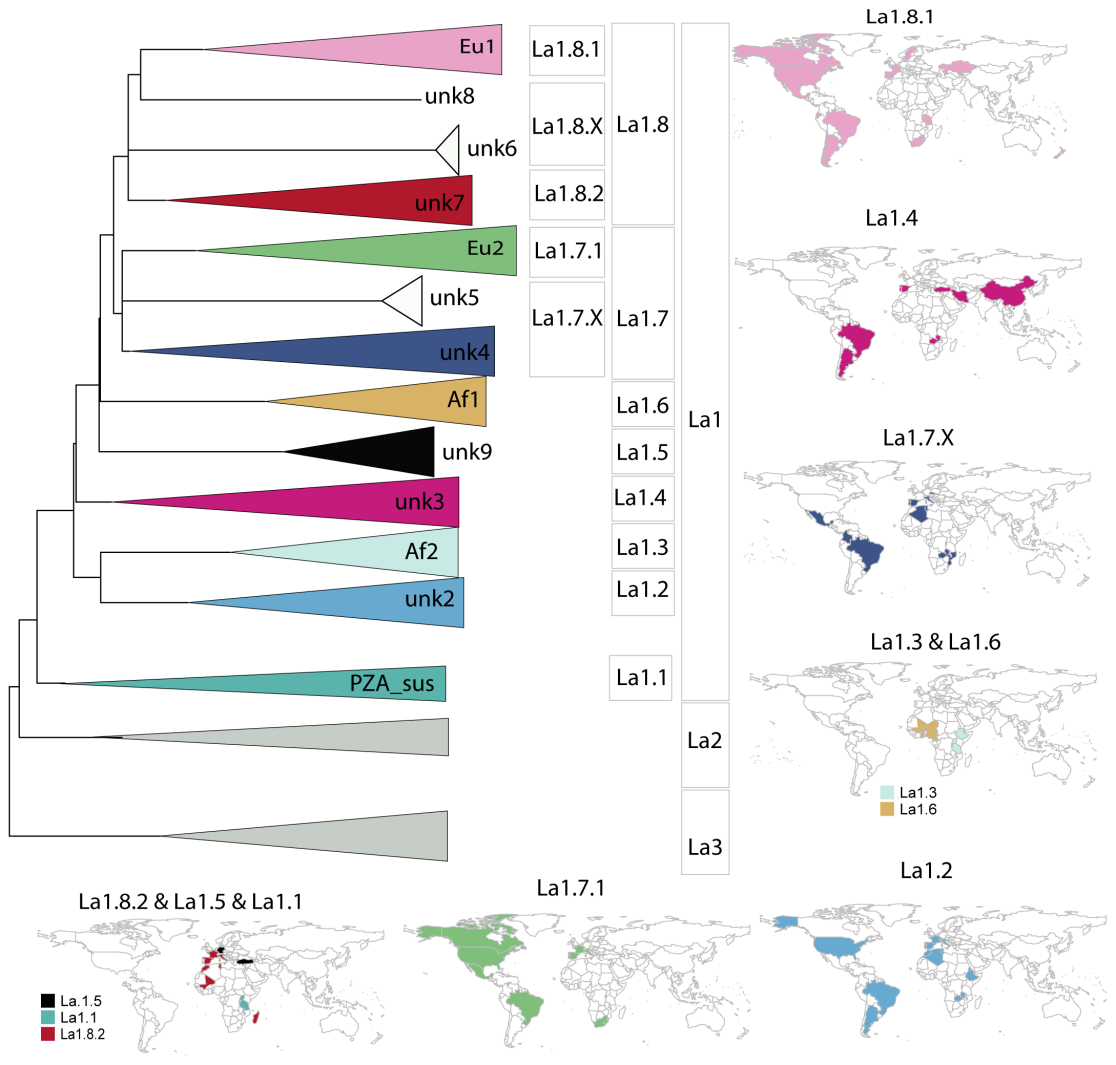
**A**) Schematic illustration of La1 sublineages. The length of the branch is not proportional to genetic distances. Groups without strong bootstrap support (<80) are shown as polytomies. Color codes are the same as in Figure 4. **B**) Geographic distribution of La1 sublineages informed by WGS and *in silico* spoligotype patterns (Extended data: Table 3).

***Sublineages La1.1 to La1.3.*** A sublineage classification was attributed to all well-resolved monophyletic clades showing a strong statistical support and separating deeply from the most recent common ancestor of La1 ( Figure 4 & Figure 5). This was clearly the case for the pyrazimamide-susceptible *M. bovis* ^17^, the unknown2 group ^17^ and clonal complex Af2 ^15^. We thus propose to classify these groups as sublineages La1.1, La.1.2 and La1.3, respectively ( Figure 4 & Figure 5). The shape of the pairwise SNP distances between and among these sublineages also reflects that they have diverged markedly from each other ( *Extended data*, Figure 1). Sublineage La1.2 appeared as one of the main genotypes circulating in continental Europe ( Figures 4 A & B and Figure 5). This sublineage had been recently called clonal complex European 3 (Eu3) ^36, 84^. The WGS we have obtained from cattle in Switzerland and Italy belonged to La1.2 ( Figure 4 A), as did a high proportion of genomes isolated from different host species in France ^21^. The BCG group of strains belongs to La1.2, and is closely related to a veterinarian sample from France, in line with the origin of the BCG vaccine strain in that country ^85^. Several genomes isolated in Ethiopia also belonged to La1.2 ^36^, reinforcing the notion that this sublineage has a strong presence in both Western Europe and East-Africa ^17, 36^.

***Sublineages La1.4 to La1.8.*** The topology of the phylogenetic tree suggested that the remaining extant groups of La1 were founded in many instances by very closely related ancestral populations, as shown by the very short internal branches connecting them ( Figure 4 A). This could be explained by a history of several migrations occurring more or less simultaneously, followed by rapid diversification in different parts of the world resulting in extant groups with a markedly different geographic distribution (e.g. the clonal complex Af1 and unknown9 groups, the unknown4 and unknown5 groups, and the unknown6 and unknown7 groups, Figure 4 A & Figure 5). Yet, the splits leading to the unknown3, unknown9 and clonal complex Af1 groups are well supported statistically ( Figure 4), and the distributions of their within- and between- pair-wise SNP distances differ markedly ( *Extended data*, Figure 1). Moreover, these groups also occupy different geographic regions ( Figure 5). We therefore propose to classify unknown3, unknown9 and Af1 as sublineages La1.4, La1.5 and La1.6, respectively ( Figure 4 & Figure 5). Most of our isolates from Turkey belonged to La1.4. The geographical distribution of La1.4 based on the WGS and *in silico* spoligotyping suggests a broad distribution spanning Asia, Europe and South America. As for La1.5, the WGS analysed here were isolated from several captive animal species in Germany ^20^, originally classified as group 09 ^20^, and from humans in Turkey (this study). All these genomes had the spoligotype pattern SB0989, of which we found reports only in the mentioned geographical regions and in Albania ^20, 86, 87^. As for La1.6, only few WGS were available; however, the work by Muller *et al*., ^16^, which provides a very comprehensive description of clonal complex Af1, highlighted the restriction of this group of strains to West-Africa.

The unknown4, clonal complex Eu2 and unknown5 groups form a well-supported monophyletic group. However, the relationships between these groups are unresolved ( Figure 4). Clonal complex Eu2 has diverged from the remaining strains, forming a well-supported monophyletic clade ( Figure 4). The strains classified as unknown4 form quite a diverse group, as indicated by the relatively long branches coalescing to their common ancestor and by the distribution of their within-pair-wise SNP distances ( Figure 4 & *Extended data*, Figure 1). These latter strains were mostly isolated in Brazil ^18^, France ^21^ and Germany ^20^. The modes of the pair-wise SNP distribution of unknown4 and unknown5 suggest, when compared to densely sampled groups like Eu2, that sampling might be incomplete ( *Extended data*, Figure 1). Finally, clonal complex Eu2 and unknown4 occur in Western Europe, America and Southern Africa, overlapping in their geographic distribution and possibly reflecting dispersion events between South America and Western Europe ( Figure 5). As for unknown5, only eight closely related genomes from strains isolated in Zambia were available. All eight genomes have the phylogenetically uninformative spoligotype pattern SB0120, limiting further inferences ^17^. Based on the above discussed points, we suggest to include clonal complex Eu2, unknown4 and unknown5 in one sublineage, hereafter called La1.7, to further classify clonal complex Eu2 as a subgroup within La1.7 called La1.7.1 and to classify unknown4 and unknown5 as sublineage La1.7.X ( Figure 4 & Figure 5). Further studies with better sampling of the unknown4 and 5 groups are necessary to better understand their population structure.

The remaining genomes classified as clonal complex Eu1, unknown7 and unknown6, and a single genome with an origin in Ethiopia (unknown8) ^17^, also form a well-supported monophyletic clade. But similarly to the example discussed above, the phylogenetic relationships of their ancestors are not well resolved. Clonal complex Eu1 and unknown7 each form well-supported monophyletic groups and have distinct geographic distributions. While clonal complex Eu1 has a broad distribution all around the world, strains classified as unknown7 seem much more geographically restricted ( Figure 5). As for unknown6, only seven closely related genomes were available, all corresponding to strains isolated in cervids in the USA ^66^. Unknown8 is most closely related to clonal complex Eu1, and as discussed elsewhere ^17, 36, 88^, probably belongs to a group of strains circulating in Ethiopia. We suggest classifying these groups into one sublineage, La1.8, and to further subdivide clonal complex Eu1 and the group unknown7 into La1.8.1 and La1.8.2, respectively ( Figure 4 & Figure 5). La1.8.1 is among the best characterised groups of strains within *M. bovis*, known to be prevalent in the UK and in regions of the world known to be former UK trading partners. L1.8.2 was mostly composed of WGS from isolates from France ^21^ and a few from Ethiopia ^36^. Similar spoligoytypes have been reported in Western- and Southern Europe, suggesting that this might be another common genotype circulating in continental Europe ( *Extended data*, Table 3). In addition, similar spoligotypes have been described in different African countries including Madagascar ( Figure 5). As for unknown6 and 8, we suggest a transient classification as La1.8.X which can be revised once more genomes become available. Given the bTB surveillance measurements taking place specially in Western countries, it is also possible that some of these groups are now rare or have even become extinct.

### Validation of lineage- and sublineage- specific markers using KvarQ

We identified SNPs that are specific to La1, La2 and La3 lineages and La1 sublineages, and which can be used as genotyping markers (see Methods). To ensure specificity, the resulting list of phylogenetic SNPs obtained from the 829 dataset was compared to a set of polymorphic positions (370,449) obtained from 4,742 WGS representing the genetic diversity of human-adapted lineages L1-L7 and L9 ^42^. After excluding those SNPs, occurring in at least one out of the 4,742 genomes representing human-adapted MTBC, 1,959 SNPs remained that were specific for La1, La2, La3 and the described La1 sublineages ( *Extended data*, Table 4). Thereof 87 were selected as phylogenetic markers to create a test suite for KvarQ ^52^ (See analysis code, *Extended data*). KvarQ is a user-friendly and platform-independent tool that enables scanning fastq files for a given list of SNPs, without the need for aligning sequencing reads to a reference genome or *de novo* assemblies ^52^. We validated the test suite with 2,774 WGS from Loiseau *et al.*, ^17^ not included in our initial 831 dataset and 66 WGS randomly chosen from recently published WGS isolated in Brazil and Algeria ^62– 64^. In parallel, the WGS used to validate KvarQ were aligned with respect to the genome of reference, as indicated in the Methods and used together with the WGS from the 829 dataset to infer a new phylogenetic tree. According to the KvarQ results ( *Extended data*, Table 5), all WGS belonged to one of the defined La1 sublineages, or to La2, and the visual inspection of the phylogenetic tree indicated that all lineage/sublineage assignments by KvarQ were correct.

Thus, here we provide a specific set of polymorphic positions that can be used to develop molecular assays to classify strains. These are provided in Table 4 ( *Extended data*) both as coordinates with respect to our genome of reference as well as with respect to the first position of genes. In the cases for which WGS exist, sequencing reads can be queried with a new suite of markers (See Zenodo repository) ^61^, using KvarQ and bypassing the need to run conventional alignment approaches and phylogenetic analysis for strain classification. The same markers have been implemented in TBProfiler ^89^.

## Conclusions

In recent years, several thousands of WGS became available for *M. bovis*, *M. caprae* and *M. orygis*, in particular for the former. Previous phylogenomic studies have unveiled that these pathogens, despite being associated with livestock species, exhibit a broad host species range and marked differences in the geographic distribution of various genotypes. Hypothetically, these genotypes might also differ in pathogenicity as observed in the case of the human-adapted MTBC members. As the number of WGS of livestock-associated MTBC continues to grow in public repositories, there is a need for a practical nomenclature allowing comparative analysis and hypothesis testing. After gathering several thousands of WGS and selecting representatives of different genotypes and geographic regions, we have obtained an exhaustive phylogenetic depiction of *M. bovis*, *M. caprae* and *M. orygis* as well as of the main genetic groups currently known within *M. bovis*. In analogy with the nomenclature in use by the scientific community for the human-adapted members of the MTBC, we proposed here a body of operational nomenclature hierarchically classifying genetic groups within the livestock-associated members in lineages and sublineages. This nomenclature classifies all main genetic groups that are known currently, and is flexible so as to accommodate new genetic diversity uncovered by future studies. We also provided specific marker SNPs that can be used to develop molecular assays to identify each of the lineages and sublineages proposed. Furthermore, we developed a new SNP test suite implemented in KvarQ and TBProfiler, which allows querying WGS without requiring a lot of bioinformatics expertise. 

## Data availability

### Underlying data

European Nucleotide Archive (EBI-EMBL): A new nomenclature for the livestock-associated Mycobacterium tuberculosis complex based on phylogenomics. Accession number: PRJEB46653, https://identifiers.org/ena.embl:PRJEB46653

European Nucleotide Archive (EBI-EMBL): Whole Genome sequencing (WGS) of Mycobacterium bovis spoligotype SB0120 and SB0841 isolates circulating in Italy. Accession number PRJEB46575, https://identifiers.org/ena.embl:PRJEB46575

Zenodo: A new nomenclature for the livestock-associated Mycobacterium tuberculosis complex based on phylogenomics, https://doi.org/10.5281/zenodo.5153095 ^90^

This project contains the following underlying data:

- Table 1: Accession numbers and metadata associated with the 829 WGS used of La1, La2 and La3.
- Table 2 - Accession numbers and metadata associated with the 1,221 WGS used as representatives of the whole MTBC.

### Extended data

Zenodo: A new nomenclature for the livestock-associated Mycobacterium tuberculosis complex based on phylogenomics, https://doi.org/10.5281/zenodo.5730685

This project contains the following extended data:

- Figure 1: Distribution of the raw pairwise SNP distances between and within main La1 groups.
- Table 3: Spoligotypes patterns inferred from the WGS and used to complement the geographic distribution of La1 sublineages.
- Table 4: Single nucleotide polymorphisms (SNPs) specific to livestock-associated MTBC lineages and sublineages. Coordinates based on the *M. tuberculosis* H37Rv annotation (NC_000962.3) are given (Position_ref), and the lineage and or sublineage classification (PhylogeneticSNP). Additionally, the gene-based position is indicated (Position_gene) as well as the kind of mutation based on SnpEff annotation ^50^. SNPs used to create the new KvarQ testsuite are indicated.
- Table 5: KvarQ results of lineage and sublineage typing done with the new testsuite implemented.

Analysis code available from :

- https://github.com/dbrites/LivestockAssociatedMTBC
- Archived analysis code at time of publication: DOI: 10.5281/zenodo.5730644
- License:GNU

Test suite and sublineages implementable in KvarQ ^52^ available from:

- https://github.com/dbrites/LivestockAssociatedMTBC/tree/main/KvarQ_testsuite/MTBC_animals
- Archived analysis code at time of publication: DOI: 10.5281/zenodo.5730644
- License: GNU
