# Supplementary material for: A new nomenclature for the livestock-associated Mycobacterium tuberculosis complex based on phylogenomics
Source: Open Res Eur. 2021 Dec 1;1:100. Originally published 2021 Aug 25. [Version 2] doi: 10.12688/openreseurope.14029.2 (PMC10445919; doi:10.12688/openreseurope.14029.2)
Supplement: Peer review history

# Peer review: Reviewer response for version 2

**DOI:** 10.21956/openreseurope.15458.r28121

**Published:** 2021-12-10

**Authors:** Lorraine Michelet

**Affiliations:** 1 Tuberculosis National Reference Laboratory, Laboratory for Animal Health, ANSES, Paris-Est University, Marne-la-Vallée, France

I acknowledge the authors for their responses. The animal lineages definition has been clarified and justified in the introduction.

Is the study design appropriate and does the work have academic merit?

Yes

Is the work clearly and accurately presented and does it cite the current literature?

Yes

If applicable, is the statistical analysis and its interpretation appropriate?

Not applicable

Are all the source data underlying the results available to ensure full reproducibility?

Yes

Are the conclusions drawn adequately supported by the results?

Partly

Are sufficient details of methods and analysis provided to allow replication by others?

Yes

Reviewer Expertise:

microbiology, molecular biology, phylogenetics

I confirm that I have read this submission and believe that I have an appropriate level of expertise to confirm that it is of an acceptable scientific standard.

# Peer review: Reviewer response for version 2

**DOI:** 10.21956/openreseurope.15458.r28122

**Published:** 2021-12-08

**Authors:** Liliana C. M. Salvador

**Affiliations:** 1 Department of Infectious Diseases, College of Veterinary Medicine, University of Georgia, Athens, GA, USA

I appreciate that the authors have addressed all my concerns. I have no further comments to make. Thank you.

Is the study design appropriate and does the work have academic merit?

Yes

Is the work clearly and accurately presented and does it cite the current literature?

Yes

If applicable, is the statistical analysis and its interpretation appropriate?

Not applicable

Are all the source data underlying the results available to ensure full reproducibility?

Yes

Are the conclusions drawn adequately supported by the results?

Partly

Are sufficient details of methods and analysis provided to allow replication by others?

Yes

Reviewer Expertise:

phylogenetics, phylodynamics, bioinformatics, M. bovis

I confirm that I have read this submission and believe that I have an appropriate level of expertise to confirm that it is of an acceptable scientific standard.

# Peer review: Reviewer response for version 1

**DOI:** 10.21956/openreseurope.15117.r27485

**Published:** 2021-10-13

**Authors:** Liliana C. M. Salvador

**Affiliations:** 1 Department of Infectious Diseases, College of Veterinary Medicine, University of Georgia, Athens, GA, USA

**Summary**

 Zwyer *et al. *proposed a new nomenclature for three species of the Mycobacterium tuberculosis complex *M. bovis*, *M. orygis* and *M. caprae* by combining phylogenetics with genomics.The goals of their study was to define lineages and sub-lineages based on genetic diversity patterns by analysing a subset of 830 whole genomes extracted from (and representative of) different parts of the world). The authors have found three main lineages La1, La2, and La3, representing respectively, *M. bovis*, *M. caprae* and *M. orygis*. Within La1, the authors have identified eight sublineages (La1.1-La1.8), which presented distinct geographical patterns (some restricted to an area, while others globally widespread). The authors have also found specific markers (SNPs) to molecularly characterize the different MTBC groups.

**General comments**

 This manuscript is highly relevant for the molecular characterization of MTBC and for future molecular epidemiological and evolutionary studies. There is a large need to extend the current classification system (L1-L7 involving *M. tuberculosis* and *M. africanum*) to other species of the complex and the authors did it using a dataset of 853 genomes from across the world. Overall, I find the manuscript well thought out, very relevant, and timely. I only have a few comments below regarding the analysis performed, which could impact the presented results.

**General comments/questions**

- Selection of isolates: authors mentioned that they chose 50 genomes per continent. What if the same amount of genomes was taken from an endemic place versus a non-endemic place. How does the local prevalence of the disease affect the genetic diversity that we see in the 50 isolates? Are the 50 genomes enough to characterize well the genetic diversity of a specific place?
- What is MTBC-Livestock associated? How is it defined? Are there any other MTBC species that infect livestock that are not included? Are there any “MTBC-Livestock associated” lineages that infect the same or even more other types of animals like, for example, badgers or white-tailed deer (both reservoirs for *M. bovis*)?

Is the study design appropriate and does the work have academic merit?

Yes

Is the work clearly and accurately presented and does it cite the current literature?

Yes

If applicable, is the statistical analysis and its interpretation appropriate?

Not applicable

Are all the source data underlying the results available to ensure full reproducibility?

Yes

Are the conclusions drawn adequately supported by the results?

Partly

Are sufficient details of methods and analysis provided to allow replication by others?

Yes

Reviewer Expertise:

phylogenetics, phylodynamics, bioinformatics, M. bovis

I confirm that I have read this submission and believe that I have an appropriate level of expertise to confirm that it is of an acceptable scientific standard, however I have significant reservations, as outlined above.

# Response

**Published:** 2021-11-18

**Authors:** Daniela Brites

**Affiliations:** 6220, Switzerland

We thank the reviewer for her positive feedback.

 As for the selection of isolates; the aim of this analysis was to have balanced phylogenetic inference of the total diversity of the MTBC. However, we were not so interested in maximizing genetic diversity within each lineage but rather to have an even representation of endemic and non-endemic places (which we believe to have achieved by sampling randomly each lineage in each continent) at a global scale and show that there is no reason to treat *M. bovis* differently than the human adapted lineages; at least not in what concerns the tree topology. Certainly, as very well pointed out by the reviewer, 50 genomes might not be enough to represent all diversity of *M. bovis* in Africa for instance, but that should be true also for other groups of the MTBC. As to the definition of livestock associated, a similar question was also raised by the other reviewer, revealing that we failed to pass on more clearly why we consider *M. bovis*, *M. orygis* and *M. caprae* as livestock-associated MTBC members. We have added to the Introduction a few considerations defending this point of view, which we hope addresses the reviewer’s concerns.

 At the core of considering these species as livestock-associated is the strong evidence that the evolutionary success of *M. bovis*, *M. orygis* and *M. caprae* is associated with the fact that these pathogens are able to cause sustainable infections in livestock species. The fact that these are multi-host pathogens allows them to linger in other animal reservoirs, and in more recent times (post bTB surveillance), this might play a crucial role for these lineages by avoiding local extinctions. However at a global and evolutionary scale, migration and population expansion of *M. bovis* (possibly also *M. caprae* and *M. orygis*, to be confirmed as more data becomes available) are most likely a consequence of cattle husbandry and movements. We think that distinguishes *M. bovis*, *M. orygis* and *M. caprae* from other animal MTBC members, which are also multi-host pathogens (e.g. *M. microti*).

# Peer review: Reviewer response for version 1

**DOI:** 10.21956/openreseurope.15117.r27482

**Published:** 2021-09-16

**Authors:** Lorraine Michelet

**Affiliations:** 1 Tuberculosis National Reference Laboratory, Laboratory for Animal Health, ANSES, Paris-Est University, Marne-la-Vallée, France

This publication propose a new nomenclature for livestock-associated *Mycobacterium tuberculosis* complex members, like what exist for human-adapted MTBC. The authors proposed to call the lineages corresponding to *M. bovis*, *M. caprae* and *M. orygis*, respectively La1 to La3 and subdivided La1 (M. bovis) into 8 sublineages. This work was necessary in order to facilitate phylogenetic analyzes and to be able to compare studies from different countries.

 However, I feel uncomfortable with the use of the term livestock-associated. Indeed, *M. bovis* and *M. caprae* are the main agents responsible for bovine (and caprine) tuberculosis but *M. orygis* is not. If we refer to the OIE Terrestrial Animal Health Code, bovine tuberculosis is caused particularly by *M. bovis* but also by *M. caprae* and, to a lesser extent, by *M. tuberculosis*.  I do not understand why the authors include *M. orygis *in the livestock-associated MTBC members. In this case, why do the authors not include *M. microti*, which is known to infect cattle (Jahans et al, 2004) and has recently been identified in several times in goat and cattle.

 I can understand that more WGS data are available for *M. orygis* and that allow to better defining this lineage. However, this MTBC member cannot be included as a livestock-associated. Even if few WGS data are available for *M. microti* and *M. pinnipedii*, I would have expected that the authors include these species as animal-adapted and define two other lineages for these species. The authors could provide some specific markers based on the few available genomes.

 In the introduction, I consider that the authors could have more described the previous works done on *M. bovis*, especially on the definition of four clonal complexes. These studies are the result of wide collaborations and have provided a much better understanding of the population structure of *M. bovis *before WGS era. Some other recent works have tried to defined lineages or clusters which are confirmed by this work but the parallel between them is not done. Some references are missing in this part of the introduction (Hauer et al, definition of  9 clusters).

 One aspect of the evolution of *M. bovis* is not discussed at all, it is the time. The year of isolation of each sample is an important data that is missing here. The authors suggest further studies on unknown 4, 5, 6 and 8 group to better understand their population structure. However, maybe this could not be done if these lineages have disappeared. For example, all unknown6 samples have been isolated in the 90’s expect one in 2008. It is well known that selection pressure due to the surveillance and eradication measures put in place in each country has led to a reduction in the genetic diversity of *M. bovis*. This aspect of *M. bovis* evolution could be discussed.

**Minor comments**

- Page 4 $ Population structure and genetic distance: add a space between “dataset,” and “using the R package”.
- Page 7 $ La2, or M. caprae: the authors refer twice to Figure 3 but this should be the Figure 4 (Figure 3 refer to La3 M. orygis).
- Page 12 $ The remaining genomes: there is a mistake in Spoligotypes.
- Extended table 1: some samples are in duplicate. For example, SRR7851305 rely to G47578 and G49365. Please check carefully your data.

Is the study design appropriate and does the work have academic merit?

Yes

Is the work clearly and accurately presented and does it cite the current literature?

Yes

If applicable, is the statistical analysis and its interpretation appropriate?

Not applicable

Are all the source data underlying the results available to ensure full reproducibility?

Yes

Are the conclusions drawn adequately supported by the results?

Partly

Are sufficient details of methods and analysis provided to allow replication by others?

Yes

Reviewer Expertise:

microbiology, molecular biology, phylogenetics

I confirm that I have read this submission and believe that I have an appropriate level of expertise to confirm that it is of an acceptable scientific standard, however I have significant reservations, as outlined above.

## References

[1] : Isolation of Mycobacterium microti from a male Charolais-Hereford cross. *Vet Rec* .2004;155(12) :373-4 15493609

[2] : Accurate Phylogenetic Relationships Among Mycobacterium bovis Strains Circulating in France Based on Whole Genome Sequencing and Single Nucleotide Polymorphism Analysis. *Front Microbiol* .2019;10: 10.3389/fmicb.2019.00955 955 10.3389/fmicb.2019.00955 31130937PMC6509552

# Response

**Published:** 2021-11-18

**Authors:** Daniela Brites

**Affiliations:** 6220, Switzerland

(Reviewer comments in italics)

*This publication propose a new nomenclature for livestock-associated Mycobacterium tuberculosis complex members, like what exist for human-adapted MTBC. The authors proposed to call the lineages corresponding to M. bovis, M. caprae and M. orygis, respectively La1 to La3 and subdivided La1 (M. bovis) into 8 sublineages. This work was necessary in order to facilitate phylogenetic analyzes and to be able to compare studies from different countries.*

*However, I feel uncomfortable with the use of the term livestock-associated. Indeed, M. bovis and M. caprae are the main agents responsible for bovine (and caprine) tuberculosis but M. orygis is not. If we refer to the OIE Terrestrial Animal Health Code, bovine tuberculosis is caused particularly by M. bovis but also by M. caprae and, to a lesser extent, by M. tuberculosis.  I do not understand why the authors include M. orygis in the livestock-associated MTBC members. In this case, why do the authors not include M. microti, which is known to infect cattle (Jahans et al, 2004) and has recently been identified in several times in goat and cattle.*

**Author Response**: We understand the reservations of the reviewer with respect to the livestock-associated terminology, as not much is known about the host range of *M. orygis*. Solid molecular evidence for *M. orygis *infections only started to emerge with whole-genome sequencing relatively recently, and we are in a time where new research about *M. orygis* is defying what we know about bovine TB and zoonotic TB in countries such as India, which has the biggest livestock population in the world and where cattle and humans co-exist very closely. Several recent studies suggest that *M. orygis* is a main cause of zoonotic TB in South Asian countries. That is particularly well demonstrated by the study of Duffy *et al* in India and by a series of other studies reporting infection caused by *M. orygis* in patients of South Asian origin living in low burden TB countries (cited in our study). There is also direct evidence of *M. orygis* infecting livestock species, and very importantly, transmitting within cattle herds. This is not the case for *M. microti* or *M. tuberculosis*, which can cause TB in livestock species, but are not able to maintain cycles of infection. Transmission of *M. orygis* within cattle is also supported by our genomic analysis. We think that these are enough evidence to consider that livestock species might be one of the reasons for the evolutionary success of *M.* *orygis* like for *M. bovis* but unlike *M. microti* and *M. tuberculosis*. The reviewer is right when referring to the OIE Terrestrial Animal Health Code and bovine TB. On the human TB side, the WHO has also considered until the latest report in 2020 that zoonotic TB was synonymous of infections by *M. bovis*. We think that reflects the fact that the molecular characterization of the agents of bovine TB in Asia is lagging behind. We have added now more explicitly these considerations to the introduction and thank the reviewer for pointing out that this rational was not clear in the first version of our manuscript.

*I can understand that more WGS data are available for M. orygis and that allow to better defining this lineage. However, this MTBC member cannot be included as a livestock-associated. Even if few WGS data are available for M. microti and M. pinnipedii, I would have expected that the authors include these species as animal-adapted and define two other lineages for these species. The authors could provide some specific markers based on the few available genomes.     Response: We believe that our previous points now demonstrate more clearly why we considered M. orygis as livestock associated. Our interest was to contribute to the molecular characterization of MTBC species that cause a high burden of disease, because they affect some of the most dense animal populations of the world, humans and livestock. We consider this necessary in the light of the growing numbers of WGS available. That is not the case for M. microti and M. pinnipedii. In addition, in this manuscript, we present a rational for defining lineages based in an interval of SNPs, which is consistent with work that has been previously done for the human-adapted lineages. As shown by us in a previous publication (Brites et al, 2018, Figure 5) M. microti and M. pinnipedii are separated by only 400-500 SNPs, and thus could not be classified as different lineages if we take the same thresholds as for the rest of MTBC complex. Perhaps their split as independent phylogenetic lineages has occurred more recently, or they might have different substitution rates, etc. In any case, we consider that having a better sampling of M. microti and M. pinnipedii is necessary to be able to draw more informed scenarios.*

*In the introduction, I consider that the authors could have more described the previous works done on M. bovis, especially on the definition of four clonal complexes. These studies are the result of wide collaborations and have provided a much better understanding of the population structure of M. bovis before WGS era. Some other recent works have tried to defined lineages or clusters which are confirmed by this work but the parallel between them is not done. Some references are missing in this part of the introduction (Hauer et al, definition of  9 clusters).*

**Author Response**: Thank you for this comment. We have added these considerations into the introduction. As to the recent works that have tried to define lineages or clusters based on WGS, we think the reviewer refers to Zimpel *et al* 2020 and Hauer *et al *2019. We have added to Table 1 the correspondence between the groups defined in these two studies and the sub-lineages we are proposing here. We explicitly mention this in the text. “The correspondence between the nomenclature we propose here and previously defined groups based on WGS is given in *Extended data,* Table 1”. The study of Hauer *et al* is now also mentioned in the Introduction and not only in the Results and Discussion section.

*One aspect of the evolution of M. bovis is not discussed at all, it is the time. The year of isolation of each sample is an important data that is missing here. The authors suggest further studies on unknown 4, 5, 6 and 8 group to better understand their population structure. However, maybe this could not be done if these lineages have disappeared. For example, all unknown6 samples have been isolated in the 90’s expect one in 2008. It is well known that selection pressure due to the surveillance and eradication measures put in place in each country has led to a reduction in the genetic diversity of M. bovis. This aspect of M. bovis evolution could be discuss.   Response: We agree with the reviewer and have added these points to the end of section Sublineages La1.4 to La1.8.*

***Minor comments***

- *Page 4 $ Population structure and genetic distance: add a space between “dataset,” and “using the R package”.*
- *Page 7 $ La2, or M. caprae: the authors refer twice to Figure 3 but this should be the Figure 4 (Figure 3 refer to La3 M. orygis).*
- *Page 12 $ The remaining genomes: there is a mistake in Spoligotypes.*
- *Extended table 1: some samples are in duplicate. For example, SRR7851305 rely to G47578 and G49365. Please check carefully*

**Author Response**: We thank the reviewer for the careful revision. Indeed, we inadvertently downloaded from public databases a few genomes twice. We have removed them from our alignments and redone the analysis and figure 4 and corrected tables 1 and table 2.
